# Clinical risk factors of bevacizumab-related hypertension in patients with metastatic colorectal cancer: a retrospective study

**DOI:** 10.3389/fphar.2024.1463026

**Published:** 2024-10-25

**Authors:** Zhuoling Zheng, Yihong Zhao, Jingwen Xie, Min Gao, Yiting Wang, Xiaoyan Li

**Affiliations:** ^1^ Department of Pharmacy, The Sixth Affiliated Hospital, Sun Yat-sen University, Guangzhou, Guangdong, China; ^2^ School of Pharmaceutical Sciences (Shenzhen), Sun Yat-sen University, Shenzhen, Guangdong, China

**Keywords:** bevacizumab, hypertension, risk factors, colorectal cancer, adverse effect

## Abstract

**Introduction:**

Bevacizumab, a vascular endothelial growth factor (VEGF) inhibitor, is widely used as a first-line treatment for metastatic colorectal cancer (mCRC), with hypertension being a common adverse effect. However, there is limited data on the predisposing factors contributing to bevacizumab-induced blood pressure (BP) elevation. This study aims to identify clinical risk factors associated with bevacizumab-related hypertension in patients with mCRC.

**Methods:**

This retrospective study included 178 patients treated between January and June 2020. Demographic data and medical histories were extracted from hospital electronic medical records.

**Results:**

Among the 178 patients, 54 (30.3%) developed bevacizumab-related hypertension, with a median onset time of 48 days. Univariate and multivariate analyses identified pre-existing hypertension [odds ratio (OR), 3.30; 95% confidence interval (CI), 1.56–6.99] and age ≥60 years (OR, 2.04; 95% CI, 1.00–4.17) as independent risk factors for bevacizumab-related hypertension. The area under the receiver operating characteristic (ROC) curve was 0.66 (95% CI, 0.57–0.75, *P* < 0.001). The median overall survival (OS) for the cohort was 30.53 months (95% CI, 22.23–38.84). No significant differences in OS were observed between patients with and without bevacizumab-related hypertension (31.13 vs. 27.87 months, *P* = 0.86).

**Conclusion:**

Pre-existing hypertension and age ≥60 years are significant clinical risk factors for bevacizumab-related hypertension in mCRC patients. Bevacizumab-related hypertension did not affect overall survival. Clinicians should closely monitor BP within the first 2 months of bevacizumab treatment in high-risk patients.

## Introduction

Colorectal cancer (CRC) is the third most common malignancy and the second leading cause of cancer-related death worldwide (Keum and Giovannucci, 2019). Despite advances in treatment and increased survival rates, metastatic colorectal cancer (mCRC) remains a lethal disease, with a 5-year survival rate of approximately 14% (Rumpold et al., 2020). Current guidelines suggest that chemotherapy combined with an anti-angiogenic agent, such as bevacizumab, is a potential first-line option for patients with mCRC (Benson et al., 2024; Deng, 2021; Mi et al., 2023). Bevacizumab, a monoclonal antibody targeting vascular endothelial growth factor A (VEGF-A), inhibits the binding of VEGF-A to its tyrosine kinase receptors, thus preventing angiogenesis (Dewdney et al., 2012). Inhibition of the VEGF pathway impedes tumor microvessel formation, delays tumor cell proliferation, reduces tumor stromal pressure, and enhances the delivery of cytotoxic drugs to tumor tissues (Ferrara et al., 2004).

Hypertension is a common adverse effect associated with bevacizumab. A meta-analysis reported that the incidence of all-grade hypertension in patients receiving bevacizumab is 23.6%, with 7.9% being classified as high-grade (grade 3 or 4) (Ranpura et al., 2010). Severe hypertension can lead to serious complications such as stroke, coronary artery disease, and other cardiovascular conditions (Ranpura et al., 2010). As survival rates for colorectal cancer patients improve, the management of bevacizumab-related hypertension becomes increasingly important. Moreover, recent studies suggest that hypertension may serve as a biomarker of bevacizumab efficacy and bevacizumab-induced hypertension is associated with improved progression-free survival (PFS) and overall survival (OS) compared to patients without hypertension (Cai et al., 2013; Dionisio et al., 2016).

Given the high incidence of bevacizumab-related hypertension and its potential prognostic value, it is crucial to identify risk factors and predict the occurrence of hypertension in bevacizumab-treated patients. Although some studies have suggested clinical risk factors such as age, body mass index (BMI), pre-existing hypertension, diabetes mellitus, dyslipidemia, renal disease, and tumor types (Hamnvik et al., 2015; Maitland et al., 2010), the results are inconsistent, and these risk factors have not been thoroughly explored in colorectal cancer patients. Therefore, more research is needed to identify predictive clinical indicators of bevacizumab-related hypertension in a broader population of colorectal cancer patients.

This study conducts a retrospective analysis of mCRC patients to evaluate bevacizumab-related hypertension. The study aims to identify demographic and clinical characteristics associated with an increased risk of hypertension in patients receiving bevacizumab. Additionally, we examine whether bevacizumab-induced hypertension is associated with OS in mCRC patients. These findings provide clinicians with valuable information to prevent and manage bevacizumab-related hypertension.

## Materials and methods

### Study population

This was a single-site, observational, and retrospective analysis. The inclusion criteria were patients with mCRC receiving bevacizumab and who had complete medical records. For patients who had pre-existing hypertension, blood pressure (BP) should be controlled stably within 2 months before bevacizumab treatment. Bevacizumab was administered intravenously at a dose of 5 mg/kg every 2 weeks in combination with chemotherapy. Exclusion criteria included patients with severe underlying medical conditions, concurrent diagnoses of other tumors, previous treatment with other VEGF inhibitors, or those who received fewer than three cycles of bevacizumab. Ethical approval was obtained from the Sixth Affiliated Hospital Ethics Committee of Sun Yat-sen University, Guangzhou, China. The study was registered in the Chinese Clinical Trial Registry (Registration Number: ChiCTR2200061510).

### Data collection

Demographic and administrative data, along with patient medical history and co-morbidities, were obtained from hospital electronic medical records for all patients receiving bevacizumab. Information on the total bevacizumab dose and concomitant chemotherapies was also recorded. Additional data collected included cancer diagnosis (TNM stage), serum sodium and potassium levels, and fasting blood glucose levels before the first bevacizumab treatment. BP measurements from 2 months before to 6 months after the initial bevacizumab dose were also retrieved. Survival data were collected from an IRB-approved prospectively maintained colorectal cancer database of the Sixth Affiliated Hospital, Sun Yat-sen University.

### Outcome definition

The primary outcome was bevacizumab-related hypertension. Hypertension was graded according to the National Cancer Institute’s Common Terminology Criteria for Adverse Events (version 5), with clinically relevant hypertension defined as grade 3 or higher. Bevacizumab-related hypertension was identified based on specific criteria for both patients with and without pre-existing hypertension. For patients without prior hypertension, post-bevacizumab hypertension was determined if any of the following occurred: discontinuation of bevacizumab due to hypertension or new diagnosis of hypertension after bevacizumab administration, initiation of antihypertensive medication, or a systolic BP of ≥160 mmHg or diastolic BP of ≥100 mmHg within 6 months after bevacizumab treatment. In patients with pre-existing hypertension, post-bevacizumab hypertension was defined by an increase in the dose or change in the type of antihypertensive medication, or if systolic BP reached ≥160 mmHg or diastolic BP reached ≥100 mmHg within 6 months of bevacizumab treatment, provided their BP had been stable for at least 2 months before starting therapy. The secondary outcome was OS, defined as the time from the start of bevacizumab therapy to the patient’s death or the last follow-up visit.

### Statistical analysis

We previously observed that approximately 30% of colorectal cancer patients receiving bevacizumab therapy developed bevacizumab-related hypertension. With a two-tailed alpha of 0.05 and a power of 0.80, this study was designed to detect differences in the incidence of hypertension in various risk factor groups (20% vs. 40%). Based on these parameters, a sample size of 156 patients was required. Ultimately, 178 patients were recruited. The statistical power was calculated using the G*Power software (version 3.1.9.7).

Patient characteristics were reported as mean ± standard deviation (SD), median (interquartile range), or number (percentage). The patients were categorized into two groups: those with bevacizumab-related hypertension and those without. Group differences were assessed using the χ^2^ test for categorical variables and the *t*-test or the Mann-Whitney *U* test for continuous variables. Logistic regression was used for the multivariable analysis, and the receiver operating characteristic (ROC) curve was used to evaluate the performance of the final multivariate model. Survival distributions were estimated using the Kaplan-Meier method, and OS was compared between the two groups using Cox regression analysis. All statistical analyses were conducted with SPSS version 26 (IBM Corp, Armonk, NY, United States), and statistical significance was defined as a two-tailed *P*-value of <0.05.

## Results

### Population characteristics

**FIGURE 1:**
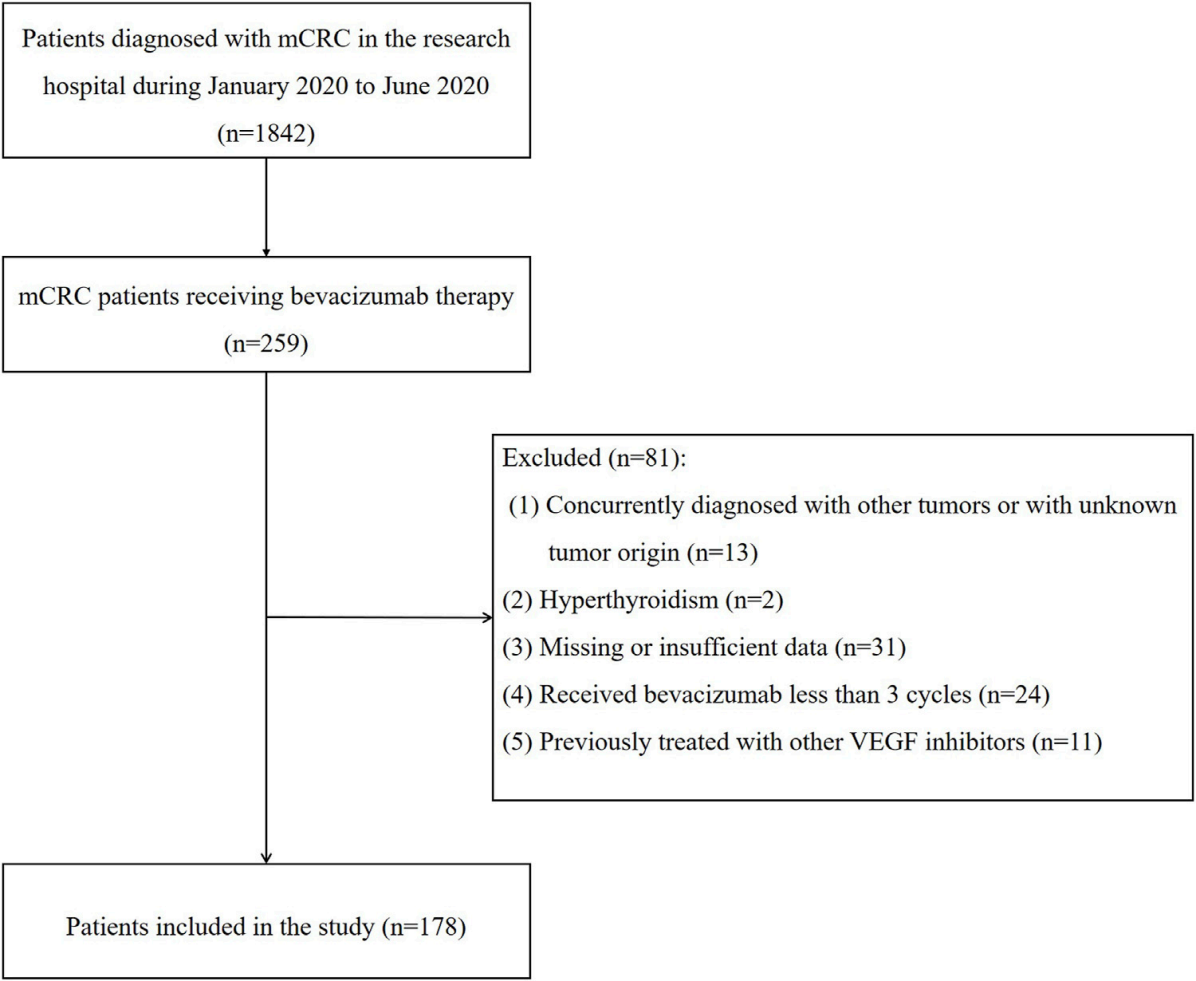
Patients’ selection process for the retrospective study.

From January 2020 to June 2020, an initial query of electronic medical records identified 1,842 patients diagnosed with mCRC, of which 259 were receiving bevacizumab therapy. After a thorough review, 81 patients were excluded, leaving 178 for analysis (Figure 1). Baseline patient characteristics are presented in Supplementary Table S1. The median age of the patients was 52.75 years (range 19–82 years), with 32.02% being 60 years or older. Of the 178 patients, 97 (54.50%) were male, and 81 (45.50%) were female. Most patients (166, 93.26%) were diagnosed with stage IV CRC before starting bevacizumab. Regarding hypertension, 43 patients (24.16%) had pre-existing hypertension, of whom 22 (51.16%) were on antihypertensive medication. The most prescribed antihypertensive agents were calcium channel blockers, such as nifedipine, used by 51.61% of these patients.

### Factors associated with bevacizumab-related hypertension

Supplementary Table S2 presents the characteristics of bevacizumab-related hypertension among study patients. Of the 178 patients, 54 (30.33%) developed bevacizumab-related hypertension. The median time to hypertension onset was 48.00 days, while the median time to maximum systolic and diastolic BP was 111.50 and 105.50 days, respectively. Following the onset of hypertension, one-third of the patients (18, 33.33%) added an antihypertensive medication. Similar to the treatment of patients with pre-existing hypertension, the majority of patients (31, 57.41%) with bevacizumab-related hypertension were treated with calcium channel antagonists.

**FIGURE 2:**
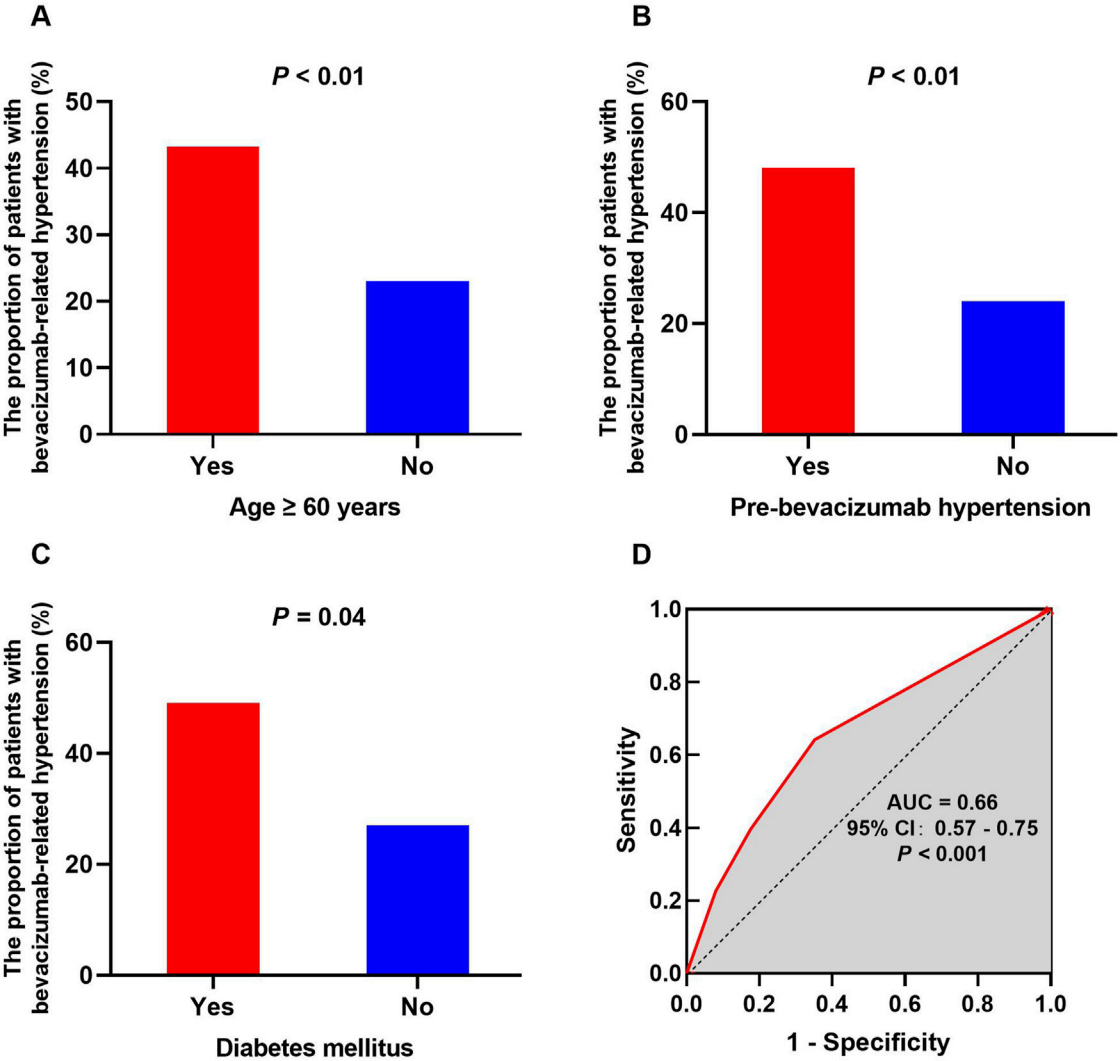
Potential risk factors for bevacizumab-related hypertension and the ROC curve of the prediction model. The influence of risk factors on the occurrence of hypertension exhibits notable variations, encompassing age ≥ 60 years **(A)**, pre-bevacizumab hypertension **(B)**, and diabetes mellitus **(C)**. The ROC curve for the final predictive model demonstrates an AUC of 0.66 **(D)**.

Univariate analyses were performed to identify possible risk factors for bevacizumab-related hypertension to examine the association between patient characteristics and hypertension (Table 1). Age ≥60 years [odds ratio (OR) = 2.48, *P* < 0.01, Figure 2A], pre-existing hypertension before bevacizumab treatment (OR = 3.37, *P* < 0.01, Figure 2B), and diabetes mellitus (OR = 2.44, *P* = 0.04, Figure 2C) were significantly associated with an increased risk of bevacizumab-related hypertension. In contrast, sex, BMI ≥ 25, smoking, and co-administration of atropine, irinotecan, or capecitabine did not have a significant effect on the incidence of bevacizumab-related hypertension. Although irinotecan co-administration was not significantly associated with bevacizumab-related hypertension, it showed a trend toward significance (OR = 0.58, *P* = 0.10). All significant risk factors met the statistical power of 0.80.

**TABLE 1:** Association between clinical factors and bevacizumab-related hypertension.

Clinical factors	Hypertension incidence (%)	Univariate analyses		Power	Multivariable analysis	
		OR [95% CI]	P		OR [95% CI]	*P*
BMI ≥25	44.4	2.08 [0.90–4.80]	0.08	0.99	—	0.18
Diabetes	48.0	2.44 [1.03–5.77]	0.04	0.99	—	0.16
Pre-existing hyperglycemia	40.9	1.89 [0.93–3.84]	0.08	0.91	—	0.40
Pre-bevacizumab hypertension	51.2	3.37 [1.65–6.91]	<0.01	0.99	3.30 [1.55–7.00]	<0.01
Age ≥ 60	43.9	2.48 [1.27–4.84]	<0.01	0.99	2.04 [1.00–4.17]	0.04
Smoking	33.3	1.16 [0.28–4.81]	1.00	0.10		
Alcohol	33.3	1.15 [0.10–12.97]	1.00	0.08		
Co-medication with atropine	26.7	0.66 [0.34–1.25]	0.20	0.60		
Co-medication with irinotecan	25.7	0.58 [0.30–1.11]	0.10	0.87	—	0.38
Co-medication with capecitabine	33.9	1.25 [0.63–2.48]	0.52	0.18		
Gender (male vs. female)	29.6% vs. 30.9	1.06 [0.56–2.02]	0.85	0.06		
Antihypertensive drugs before medication						
ACEI	50.0	2.32 [0.14–37.80]	0.52	0.99		
ARB	40.0	1.57 [0.43–5.82]	0.74	0.64		
β-blocker	0.0	—	1.00	—		
CCB	37.5	1.43 [0.49–4.14]	0.71	0.42		
Diuretics	50.0	2.32 [0.14–37.80]	0.52	0.99		

Note: ACEI, angiotensin-converting enzyme inhibitor; ARB, angiotensin receptor blocker; CCB, calcium channel blocker; OR, odds ratio; CI, confidence interval.

Logistic regression was used for multivariate analysis (Table 1). Based on the results of the χ^2^ test, factors with *P* ≤ 0.10 were selected for inclusion in the logistic regression model, including BMI ≥ 25, history of diabetes, pre-existing hyperglycemia, pre-bevacizumab hypertension, age ≥ 60, and co-administration of irinotecan. In the final model, pre-bevacizumab hypertension (OR = 3.30, *P* < 0.01) and age ≥60 (OR = 2.04, *P* = 0.04) were identified as independent risk factors for bevacizumab-related hypertension. The ROC curve was used to evaluate the predictive value of the model, with an area under the ROC curve (AUC) of 0.66 (95% CI, 0.57–0.75, *P* < 0.001, Figure 2D).

### Relationship between bevacizumab-related hypertension and OS

**FIGURE 3:**
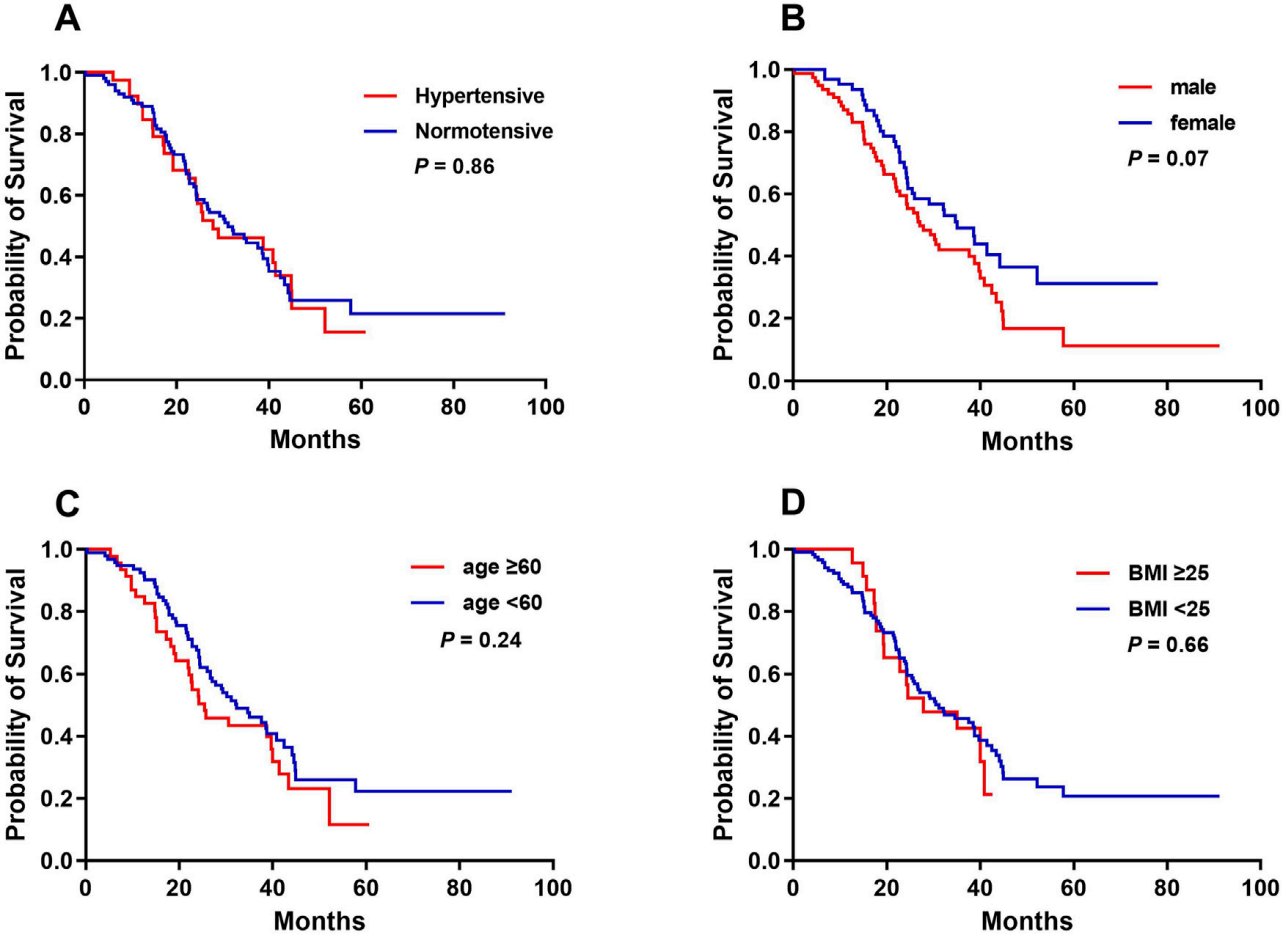
Kaplan-Meier analysis of overall survival curves in accordance with bevacizumab-related hypertension **(A)**, sex **(B)**, age **(C)**, and BMI **(D)**.

The median OS for the cohort was 30.53 months (95% CI, 22.23–38.84). There were no significant differences in OS between patients with and without bevacizumab-related hypertension (31.13 vs. 27.87 months, *P* = 0.86). Additionally, sex, age ≥60 years, and BMI ≥ 25 had no significant impact on OS (Figure 3).

## Discussion

Bevacizumab, a monoclonal antibody targeting VEGF, is widely used as a first-line treatment for mCRC. One of the typical adverse reactions to bevacizumab is hypertension. Current guidelines recommend assessing BP before initiating bevacizumab treatment, and it is suggested that patients with a clinic BP of <160/100 mmHg can safely start therapy (Plummer et al., 2019). Although hypertension is often asymptomatic, unmanaged hypertension can lead to serious cardiovascular complications, such as reversible posterior leukoencephalopathy syndrome (Ozcan et al., 2006) and subarachnoid hemorrhage (Baizabal-Carvallo et al., 2010). Despite the prevalence of bevacizumab-related hypertension, its risk factors have not been fully explored in colorectal cancer patients. This study aimed to address this gap by conducting a retrospective analysis of CRC patients receiving bevacizumab and identified pre-existing hypertension and age ≥60 years as significant risk factors for developing bevacizumab-related hypertension.

To assess bevacizumab-related hypertension, we collected data on antihypertensive medication use and BP from 2 months before to 6 months after the first dose of bevacizumab. Given the half-life of bevacizumab of 20 days, with typical administration intervals of 2–3 weeks, bevacizumab-induced hypertension is dose-dependent (Zhu et al., 2007). A study by Maitland et al. (2010) confirmed that BP elevation typically occurs within the first treatment cycle, usually within 14 days of administration. Additionally, Hamnvik et al. (2015) reported a median time to high-grade hypertension of 29 days. Thus, the 6-month observation period used in this study was considered appropriate. We observed a bevacizumab-induced hypertension incidence rate of 30.33%. In comparison, a meta-analysis by Ranpura et al. (2010) reported an incidence of high-grade hypertension (grade 3–4) between 1.8% and 22%, slightly lower than the current study findings. The discrepancy may be because Ranpura’s analysis was based on randomized clinical trials. At the same time, the present study is a retrospective analysis conducted in a real-world setting, which may explain the higher incidence of hypertension.

Pre-existing hypertension is associated with the development of bevacizumab-related hypertension (Hamnvik et al., 2015; Isobe et al., 2014). Consistent with previous research (Wicki et al., 2014; Hamnvik et al., 2015), the present study found that pre-bevacizumab hypertension was significantly related to the occurrence of bevacizumab-related hypertension, further emphasizing pre-existing hypertension as a significant risk factor. However, the use of antihypertensive medication and the specific type of treatment were not associated with the development of bevacizumab-related hypertension. Therefore, prophylactic administration of antihypertensive drugs is not recommended for patients with normal BP before bevacizumab treatment. Patients with underlying hypertension are more susceptible to post-bevacizumab hypertension, warranting closer monitoring. For colorectal cancer patients with a history of hypertension, BP should be carefully monitored after bevacizumab initiation. Regular BP evaluation is crucial during the initiation, maintenance, and titration of bevacizumab therapy. Out-of-office BP monitoring, such as home BP assessments, is an effective strategy, and patients should be encouraged to monitor their BP at home regularly (Syrigos et al., 2011).

In this study, age ≥60 years was significantly associated with an increased risk of bevacizumab-induced hypertension. This finding is consistent with previous research, which also identified age as a risk factor for BP elevation during anti-VEGF therapy (Li et al., 2018). However, another study did not report age as a significant factor (Wicki et al., 2014). These discrepancies may arise from differences in age stratification methods between studies. Cancer incidence and prevalence increase with age (Siegel et al., 2022), being lowest in individuals aged 35–39 years and peaking in the 80–84 age group. In China, the incidence of colorectal cancer begins to increase markedly at the age of 50 years (Cao et al., 2020). Chronic medical conditions are also more common in older adults, who may have diminished organ function. A review reported that the incidence of hypertension in mCRC patients was 11% in those aged 65–75 years, compared to 29% in patients over 75 (Raman et al., 2007). Thus, older age may be a risk factor for bevacizumab-induced hypertension. However, bevacizumab has significantly improved PFS and OS in elderly patients with mCRC (Pinto et al., 2017). While older age is not an absolute contraindication to the use of bevacizumab, elderly patients often present with unique challenges, such as comorbidities and polypharmacy (Aprile et al., 2013). Therefore, careful benefit-risk evaluation and regular out-of-office BP monitoring are essential for elderly mCRC patients undergoing bevacizumab treatment.

Diabetes is another crucial risk factor for hypertension, with individuals in the pre-diabetes stage having 1.5 times the risk of hypertension compared to those with normal glucose metabolism (Tsimihodimos et al., 2018). High blood glucose levels before antitumor therapy may influence the incidence of bevacizumab-induced hypertensive side effects. In this study, diabetes mellitus significantly increased the probability of bevacizumab-related hypertension; however, it was not included in the final logistic regression model, possibly because its effect on BP was less significant than that of age and pre-bevacizumab hypertension. Since hypertension and diabetes mellitus often co-develop, patients with diabetes should be closely monitored for hypertension when receiving bevacizumab to enable early detection and management.

In addition to the risk factors mentioned above, combination therapy may also contribute to the development of bevacizumab-induced hypertension. In this study, patients who received irinotecan in combination with bevacizumab exhibited a trend toward a lower incidence of hypertension. This effect may be related to the ability of irinotecan to increase the acetylcholine concentration at synapses (Iihara et al., 2019). Acetylcholine reduces heart rate and cardiac output, thus lowering blood pressure, acting similarly to beta-blockers, which may reduce the incidence of hypertensive side effects (Blandizzi et al., 2001). However, irinotecan is often administered with atropine, which blocks acetylcholine receptors and may counteract the hypotensive effects of irinotecan (de Man et al., 2018). Despite this, irinotecan showed a tendency to reduce the incidence of bevacizumab-induced hypertension in this study. More research involving larger patient populations is needed to better understand the effect of irinotecan on blood pressure when combined with bevacizumab.

Several retrospective studies have suggested a correlation between bevacizumab-induced hypertension and clinical outcomes in CRC patients (Scartozzi et al., 2009; Lombardi et al., 2022; Osterlund et al., 2011). However, in this study, no significant association was found between OS and bevacizumab-induced hypertension. The use of hypertension as a potential biomarker for clinical outcomes is complex. Generally, increases in blood pressure are moderate, and hypertensive crises are rare. Therefore, it is not yet clear the degree of BP elevation that correlates with prognosis remains unclear. This study examined grade 3–4 hypertension caused by bevacizumab and did not observe significant effects on OS. In contrast, other studies have demonstrated prognostic significance for patients with any grade of hypertension (Osterlund et al., 2011). Clinically important cut-off values for percentage increases in BP still need to be defined. Although biomarkers for OS are important, as mCRC survival rates continue to improve, prediction and management of potential long-term side effects such as hypertension are becoming increasingly critical.

This study has several limitations. First, the sample size was relatively small, and no additional cardiac or renal events were observed in the group requiring the addition of antihypertensive medications. Consequently, future studies should validate these results in a larger population. Additionally, the study lacks comprehensive therapeutic assessment indices, such as PFS and the objective response rate, making it difficult to determine the association between bevacizumab-related hypertension and prognosis. Finally, the predictive model developed in this study only included clinical factors and required further refinement. Future research should also consider the potential influence of genetic factors on the development of bevacizumab-induced hypertension.

## Conclusion

Pre-existing hypertension and age ≥60 were identified as clinical risk factors for bevacizumab-related hypertension in mCRC patients. The median time to the onset of hypertension was 48 days, and no correlation was found between bevacizumab-related hypertension and OS. Clinicians should closely monitor blood pressure during the first 2 months of bevacizumab treatment, particularly in high-risk patients. More studies with larger sample sizes are needed to validate these findings and explore the possible association between bevacizumab-induced hypertension and patient prognosis.

## Data Availability

The raw data supporting the conclusions of this article will be made available by the authors, without undue reservation.
