# Supplementary material for: Clinical risk factors of bevacizumab-related hypertension in patients with metastatic colorectal cancer: a retrospective study
Source: Front Pharmacol. 2024 Oct 25;15:1463026. doi: 10.3389/fphar.2024.1463026 (PMC11543948; doi:10.3389/fphar.2024.1463026)
Supplement: Supplementary file 1 [file Table1.docx]

Supplementary Material

# Supplementary Tables

**Supplementary Table 1**. Baseline characteristics of the patients

| Characteristics | No. of patients |
| --- | --- |
| Age (years) | 52.75 ± 12.88 |
| age ≥ 60 years | 57 (32.02%) |
| Gender |  |
| Male | 97 (54.50%) |
| Female | 81 (45.50%) |
| Hight (cm) | 163.13 ± 8.43 |
| Weight (kg) | 59.01 ± 10.11 |
| BMI (kg/m^2^) | 22.10 ± 2.92 |
| BMI ≥ 25 | 27 (15.20%) |
| Past medical history and co-morbidities | 61(34.27%) |
| Tumor TMN stages |  |
| II | 2 (1.12%) |
| III | 10 (5.62%) |
| IV | 166 (93.26%) |
| Biochemical values |  |
| Serum sodium (mmol/L) | 139.43 ± 2.75 |
| Serum potassium (mmol/L) | 3.99 ± 0.40 |
| Blood sugar (mmol/L) | 6.45 ± 2.95 |
| Pre-bevacizumab hypertension | 43 (24.16%) |
| Number of antihypertensive drugs before bevacizumab medication |  |
| 0 | 21 (48.84%) |
| 1 | 14 (32.56%) |
| 2 | 8 (18.60%) |
| Types of antihypertensive drugs before bevacizumab medication |  |
| ACEI | 2 (4.65%) |
| ARB | 10 (23.26%) |
| β-blocker | 1 (2.33%) |
| CCB | 16 (37.21%) |
| Diuretics | 2 (4.65%) |

Note: Values are mean ± SD, n (%), or median (interquartile range). ACEI, angiotensin-converting enzyme inhibitors. ARB, angiotensin receptor blocker. CCB, calcium channel blocker.

**Supplementary Table 2**. Changes in blood pressure and antihypertensive drugs in patients after receiving bevacizumab

| Characteristics | No. of patients |
| --- | --- |
| Bevacizumab-related hypertension | 54 (30.33%) |
| Non-post-bevacizumab hypertension groups | 32 (59.26%) |
| Critical BP | 25 (78.13%) |
| BP medication | 17 (53.13%) |
| Diagnosed with hypertension | 3 (9.38%) |
| Post-bevacizumab hypertension groups | 22 (40.74%) |
| Critical BP in those with stable control | 7 (31.82%) |
| Increased dose of antihypertensive drugs | 5 (22.73%) |
| Increased types of antihypertensive drugs | 14 (63.64%) |
| The median time to hypertension (days) | 48.00 [17.00, 104.50] |
| The time to maximum systolic BP (days) | 111.50 [69.75, 153.00] |
| The time to maximum diastolic BP (days) | 105.50 [63.75, 155.00] |
| Number of antihypertensive drugs after bevacizumab treatment |  |
| -1 | 2 (3.70%) |
| 0 | 21 (38.89%) |
| 1 | 18 (33.33%) |
| 2 | 10 (18.52%) |
| 3 | 3 (5.56%) |
| Types of antihypertensive drugs |  |
| ACEI | 2 (3.70%) |
| ARB | 10 (18.52%) |
| β-blocker | 31 (57.41%) |
| CCB | 7 (12.96%) |

Note: Values are mean ± SD, n (%), or median (interquartile range). BP, blood pressure. ACEI, angiotensin-converting enzyme inhibitors. ARB, angiotensin receptor blocker. CCB, calcium channel blocker.
